# The prognosis of hepatoid adenocarcinoma of the stomach: a propensity score-based analysis

**DOI:** 10.1186/s12885-020-07031-9

**Published:** 2020-07-17

**Authors:** Kai Zhou, Anqiang Wang, Sheng Ao, Jiahui Chen, Ke Ji, Qifei He, Xin Ji, Xiaojiang Wu, Ji Zhang, Zhongwu Li, Zhaode Bu, Jiafu Ji

**Affiliations:** 1 Department of Gastrointestinal Surgery, Key Laboratory of Carcinogenesis and Translational Research (Ministry of Education), Peking University Cancer Hospital & Institute, No. 52 Fucheng Road, Haidian District, Beijing, 100142 China; 2 Department of Gastrointestinal Surgery, Peking University Shenzhen Hospital, Shenzhen, 518036 Guangdong China; 3 Department of Pathology, Key Laboratory of Carcinogenesis and Translational Research (Ministry of Education), Peking University Cancer Hospital & Institute, Beijing, 100142 China

**Keywords:** Hepatoid adenocarcinoma of the stomach, Non-hepatoid adenocarcinoma of the stomach, Overall survival, Prognosis

## Abstract

**Background:**

To investigate whether there is a distinct difference in prognosis between hepatoid adenocarcinoma of the stomach (HAS) and non-hepatoid adenocarcinoma of the stomach (non-HAS) and whether HAS can benefit from radical surgery.

**Methods:**

We retrospectively reviewed 722 patients with non-HAS and 75 patients with HAS who underwent radical gastrectomy between 3 November 2009 and 17 December 2018. Propensity score matching (PSM) analysis was used to eliminate the bias among the patients in our study. The relationships between gastric cancer type and overall survival (OS) were evaluated by the Kaplan-Meier method and Cox regression.

**Results:**

Our data demonstrate that there was no statistically significant difference in the OS between HAS and non-HAS {K-M, P = log rank (Mantel-Cox), (before PSM P = 0.397); (1:1 PSM P = 0.345); (1:2 PSM P = 0.195)}. Moreover, there were no significant differences in the 1-, 2-, or 3-year survival rates between patients with non-HAS and patients with HAS (before propensity matching, after 1:1 propensity matching, and after 1:2 propensity matching).

**Conclusion:**

HAS was generally considered to be an aggressive gastric neoplasm, but its prognosis may not be as unsatisfactory as previously believed.

## Background

Gastric carcinoma (GC) is not only the second most common cancer but also the third leading cause of death in China [1, 2], which poses a great threat to people’s health in China [3]. Although the incidence rate of GC has been declining steadily with the improvement of heath standards, nutrition levels and radical treatment of *Helicobacter pylori*, the long-term survival is far from satisfactory [4–6]. Rare types of cancer without standard treatment modalities partly contribute to the adverse outcomes of GC. As a rare type of GC [7, 8], hepatoid adenocarcinoma of the stomach (HAS) is a special type of extrahepatic carcinoma characterized by the histological resemblance to hepatocellular carcinoma [9, 10].

In 1970, Bourreille first reported one case of α-fetoprotein-producing gastric carcinoma with liver metastasis [11]. Later, Ishikura et al. named it “hepatoid adenocarcinoma of the stomach” for primary GC [12, 13]. It was reported that this rare type of GC accounts for 0.38 to 1% of GC. In addition to similar clinical features, such as occurring mainly in elderly and male patients [7], HAS was found to be accompanied by a higher rate of lymph node and liver metastasis in comparison with GC [14, 15]. Additionally, more than 80% of HAS patients had elevated serum α-fetoprotein (AFP) levels [14, 16]. Considering the higher rate of metastasis, the prognosis of HAS has been widely reported to be inferior to that of non-HAS [17]. To the best of our knowledge, however, most studies have been limited to case reports or case series [18]. Therefore, a more systematic study with more cases is especially meaningful for the prognostic exploration of HAS.

In our study, to explore the prognosis of HAS and whether HAS can benefit from radical surgery, we conducted propensity score-based analyses on a larger number of patients with GC.

## Methods

### Patients

Patients of 797 who underwent radical surgical resection for gastric carcinoma at the Peking University Cancer Hospital between 3 November 2009 and 17 December 2018 were considered for inclusion in the study. Patients with GC were diagnosed by gastroscopy, biopsy and computed tomography. GC patients with sufficient clinicopathological information were included in our research. However, patients without radical surgery and who were diagnosed with non-adenocarcinoma were excluded. For advanced gastric cancer (including non-HAS and HAS), if there was no distant metastasis or invasion of surrounding organs, D2 lymphadenectomy is recommended, which is performed by experienced doctors.

We collected clinical information, including sex, age, tumor location, surgery type, and levels of carcinoembryonic antigen (CEA) and carbohydrate antigen 19–9 (CA199). Pathological features such as vascular invasion, TNM stage (American Joint Committee on Cancer (AJCC), 7th edition), immunohistochemistry results and neoadjuvant chemotherapy were also gathered. The basic clinical characteristics were listed in (Additional file 1: Table 1).

### The diagnose of patients

Before surgery, pelvic and abdominal contrast-enhanced computed tomography (CT) was used to check patients’ condition of local lymph nodes and surrounding organs and determine the lesion area of GC, while gastroscopy biopsy was performed to determine the pathological type at the same time. The gastroscopy biopsy and (or) surgical specimens of HAS both have different percentages of HAS cell components. Once the pre-operative and postoperative specimens were found with adenocarcinoma, hepatocellular carcinoma or the sole hepatocyte-like regions in morphology, which would be added to examine several immune-histochemical markers, such as AFP [19], Glypican-3(GPC-3), SALL4 and Hap-Par 1 [7, 20]. If the immunohistochemical marker AFP was positive, we preliminarily considered the diagnosis of a patient’s disease as HAS. Under these circumstances, most lesions contained aberrant hepatocellular differentiation [21]. The neoplastic tissue of HAS had other than a trabecular pattern with a round to ovoid nuclei, some existed intranuclear pseudoinclusions, and some with large cells of eosinophilic cytoplasm as well as prominent nucleoli [14]. The gastroscopy biopsy and (or) surgical specimens of HAS had different HAS cell component percentages [7]. Therefore, the definitive diagnosis of HAS depended on the histomorphological features plus immunophenotypical evidence [19].

Two pathologists assessed AFP staining based on the percentage of stained celled and staining density. The percentage score of stained cells was divided into three groups: 0 for unstained cells, 1 for 1–50% stained cells, and 2 for 51–100% stained cells. The staining density ranged from 0 to 3: 0 for intense, 1 for mild, 2 for moderate, and 3 for staining [7].

### Follow-up visits

We completed the follow-up at our hospital by telephone. The status of all patients was assessed every 3 to 6 months during follow-up. We routinely checked chest and abdominal computed tomography (CT), tumor markers (CEA, CA199, carbohydrate antigen 242(CA242), carbohydrate antigen 724 (CA724)). Liver-specific contrast-enhanced Magnetic Resonance Imaging (MRI) (the result of this check was presented as multiple lesions in the liver, which was typically characterized by the bovine eye sign), positron emission tomography-computed tomography (PET-CT) and other examinations were considered to be checked according to the special situations of patients. Overall survival (OS) time was recorded during the time from the date of surgery to the date of death of cancer or the date of the last follow-up. The follow-up period lasted three years.

### Propensity score analysis (PSM)

To accurately analyzed the prognosis of HAS, we used propensity score matching to balance out the bias between HAS and non-HAS patients. The propensity score of all patients was determined by using the chi-square and Mann-Whitney U tests (Table 1). According to a 0.02 caliper width, one-to-one nearest-neighbour matching was carried out. One-to-two nearest-neighbour matching was performed with a 0.05 caliper width.

**Table 1:** Clinicopatholodical characteristics of patients with HAS and Non-HAS treated with radical gastrectomy

Factors	Before propensity matching			After 1:1propensity matching			After 1:2 propensity matching		
	Non-HAS	HAS	P value*	Non-HAS	HAS	P value	Non-HAS	HAS	P value
	(*n* = 711)	(*n* = 73)		*n* = 72	*N* = 72		*n* = 110	*n* = 55	
	No. (%)	No. (%)		No. (%)	No. (%)		No. (%)	No. (%)	
Sex (M/F)	509/202	60/13	0.053	60/12	59/13	0.826	87/23	44/11	0.892
Age (median) (yr))^#^	59 (24-84)	58 (26-76)	0.720	62 (31-81)	58 (26-76)	0.155	58 (36-80)	60 (26-76)	0.136
<45	78 (11.0)	7 (9.6)		3 (4.2)	7 (9.7)		12 (10.9)	5 (9.1)	
60>age≥45	278 (39.1)	32 (43.8)		26 (36.1)	31 (43.1)		50 (45.5)	22 (40.0)	
≥60	355 (49.9)	34 (46.6)		43 (59.7)	34 (47.2)		48 (43.6)	28 (50.9)	
Location^a^			0.364			0.640			0.785
U	217 (30.5)	25 (34.2)		19 (26.4)	24 (33.3)		39 (35.5)	19 (34.5)	
M	126 (17.7)	9 (12.3)		11 (15.3)	9 (12.5)		11 (10.0)	7 (12.7)	
L	360 (50.6)	39 (53.4)		42 (58.3)	39 (54.2)		59 (53.6)	29 (52.7)	
T	8 (1.1)	0 (0.0)		0 (0.0)	0 (0.0)		1 (0.9)	0 (0.0)	
Surgery type			0.584			1.000			0.445
PG	9 (1.3)	2 (2.7)		0 (0.0)	1 (0.7)		1 (0.9)	2 (3.6)	
DG	350 (49.2)	39 (53.4)		42 (58.3)	40 (56.9)		55 (50.0)	30 (54.5)	
TG	349 (49.1)	32 (43.8)		30 (41.7)	31 (42.4)		53 (48.2)	23 (41.8)	
TGC	3 (0.4)	0 (0.0)		0 (0.0)	0 (0.0)		1 (0.9)	0 (0.0)	
Vascular invasion			0.328			0.074			0.315
no	325 (45.7)	29 (39.7)		18 (25.0)	28 (38.9)		49 (44.5)	20 (36.4)	
yes	386 (54.3)	44 (60.3)		54 (75.0)	44 (61.1)		61 (55.5)	35 (63.6)	
T			0.004			0.051			0.990
Tis, T0, T1, T2	171 (24.0)	21 (28.8)		18 (25.0)	20 (27.8)		34 (30.9)	17 (30.9)	
T3	260 (36.6)	39 (53.4)		26 (36.1)	37 (51.4)		51 (46.4)	26 (47.3)	
T4	280 (39.4)	13 (17.8)		28 (38.9)	15 (20.8)		25 (22.7)	12 (21.8)	
N			0.180			0.182			0.566
N0	221 (31.1)	11 (15.1)		12 (16.7)	11 (15.3)		29 (26.4)	11 (20.0)	
N1	154 (21.7)	23 (31.5)		18 (25.0)	23 (31.9)		24 (21.8)	17 (30.9)	
N2	143 (20.1)	22 (30.1)		14 (19.4)	23 (31.9)		30 (27.3)	13 (23.6)	
N3	293 (27.1)	17 (23.3)		28 (38.9)	15 (20.8)		27 (24.5)	14 (25.5)	
M						1.00			1.000
M0	711 (100)	73 (100)		69 (95.8)	70 (97.2)		109 (99.1)	54 (98.2)	
M1	0	0		3 (4.2)	2 (2.8)		1 (0.9)	1 (1.8)	
EGFR			<0.001			0.940			0.695
-	64 (9.0)	2 (2.7)		1 (1.4)	2 (2.8)		6 (5.5)	2 (3.6)	
+	259 (36.4)	6 (8.2)		16 (22.2)	7 (9.7)		10 (9.1)	7 (12.7)	
++	195 (27.4)	36 (49.3)		23 (31.9)	37 (51.4)		46 (41.8)	26 (47.3)	
+++	193 (27.1)	29 (39.7)		32 (44.4)	26 (36.1)		48 (43.6)	20 (36.4)	
Ki-67			0.003			0.648			0.741
0-25%	65 (9.1)	5 (6.8)		3 (4.2)	5 (6.9)		10 (9.1)	5 (9.1)	
26-50%	168 (23.6)	5 (6.8)		10 (13.9)	6 (8.3)		12 (10.9)	6 (10.9)	
51-75%	208 (29.3)	25 (34.2)		18 (25.0)	24 (33.3)		41 (37.3)	16 (29.1)	
76-100%	270 (38.0)	38 (52.1)		41 (56.9)	37 (51.4)		47 (42.7)	28 (50.9)	
CEA (ng/ml)			<0.001			0.384			0.266
≤5	575 (80.9)	45 (61.6)		49 (68.1)	44 (61.1)		77 (70.0)	43 (78.2)	
>5	136 (19.1)	28 (38.4)		23 (31.9)	28 (38.9)		33 (30.0)	12 (21.8)	
CA199 (u/ml)			0.026			0.347			0.716
≤37	604 (85.0)	69 (94.5)		65 (90.3)	68 (94.4)		105 (95.5)	51 (92.7)	
>37	107 (15.0)	4 (5.5)		7 (9.7)	4 (5.6)		5 (4.5)	4 (7.3)	
Her-2			0.012			0.962			0.883
-/+	533 (75.0)	43 (58.9)		43 (59.7)	43 (59.7)		74 (67.3)	35 (63.6)	
+++	54 (7.6)	10 (13.7)		10 (13.9)	9 (12.5)		10 (9.1)	6 (10.9)	
++	124 (17.4)	20 (27.4)		19 (26.4)	20 (27.8)		26 (23.6)	14 (25.5)	
neoadjuvant chemotherapy			0.005			0.441			0.880
no	628 (88.3)	56 (76.7)		52 (72.2)	56 (77.8)		93 (84.5)	46 (83.6)	
yes	83 (11.7)	17 (23.3)		20 (27.8)	16 (22.2)		17 (15.5)	9 (16.4)	

M=male, F=female ^a^Divide the major and minor curvature of the stomach into 3 equal parts, connect their corresponding points, can be divided into upper 1/3(U) middle 1/3 (M), lower 1/3 (L) and the total stomach (T) TG= total gastrectomy DG=distal gastrectomy PG=proximal gastrectomy

TGC=gastrectomy combined with visceral resection * categorical data were using the chi-square test (X ²test), and continuous data were using the Mann-Whitney U test. # median (range), and compared by non-parametric tests

### Statistical analysis

Statistical analysis was conducted by using SPSS software version 23.0 (IBM, United States). The statistical significance of categorical data was assessed by using the chi-square test (X^2^ test), and continuous data using the Mann-Whitney U test. We found that T stage (infiltration depth), EGFR, KI-76, the level of CEA and CA199, HER2 and neoadjuvant chemotherapy had statistically significant differences between HAS and non-HAS groups. According to the outcome and confounding variables to built a binary logistic regression analysis, and took stepwise regression. The variables of entering the model or having clinical significance were selected into the Covariates, elimination variables into the additional covariance of PSM. PSM effectively balanced the mixed bias of group HAS and non-HAS. We utilized the method of PSM to get two schemes of which the ratio were 1:1 and 1:2 (HAS: non-HAS), respectively. For the univariate analysis of OS, the Kaplan-Meier approach was used. For the multivariate analysis, the Cox regression was used. P < 0.05 was considered as the threshold of having statistical significance. In order to obtain a more vivid and beautiful survival analysis curve, Kaplan-Meier survival plots were made by using GraphPad Prism 5.

## Results

### Study population

**Fig. 1:**
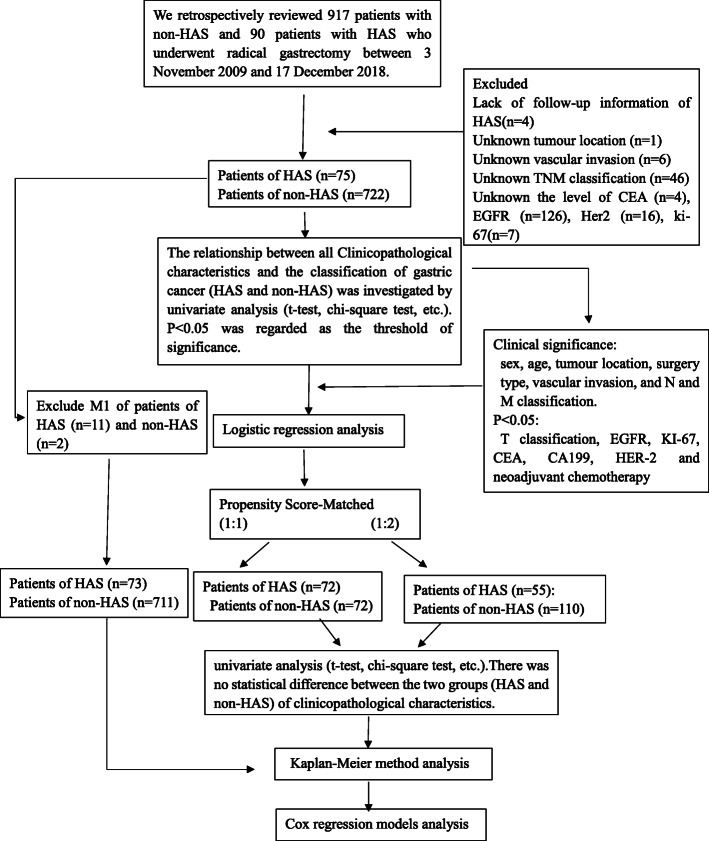
Analysis follow char

From November 2009 to December 2018, 797 patients were enrolled in our research. A total of 722 (90.6%) gastric adenocarcinoma cases (non-HAS) and 75 (9.4%) HAS cases were detected by histological morphology and immunohistochemistry. However, 11 non-HAS and 2 HAS patients hand distant metastases (M1). To reduce bias, these patients were excluded from this evaluation. Through one-to-one nearest-neighbour matching with a 0.02 caliper width, 144 patients were included for analysis, with 72 HAS and non-HAS patients each. Through one-to-two nearest-neighbour matching with a 0.05 caliper width, 165 patients were included in our study, with 110 non-HAS patients and 55 HAS patients. (Fig. 1).

### Clinicopathological characteristics

For the 797 patients, the two groups (HAS and non-HAS) were consistent in terms of sex, age, tumor location, surgery type, vascular invasion, and N (lymph node metastasis) and M stage (distant metastasis). Nevertheless, the two groups were differentially distributed in terms of T stage, EGFR, KI-67, CEA, CA199, HER-2 and neoadjuvant chemotherapy. One-to-one and one-to-two nearest-neighbour matching were used to generate 144 and 165 patients from the two groups, respectively. They showed no significant bias in clinicopathological characteristics. (Additional file 1: Table 1).

### Survival among all patients and propensity-matched pairs

**Fig. 2:**
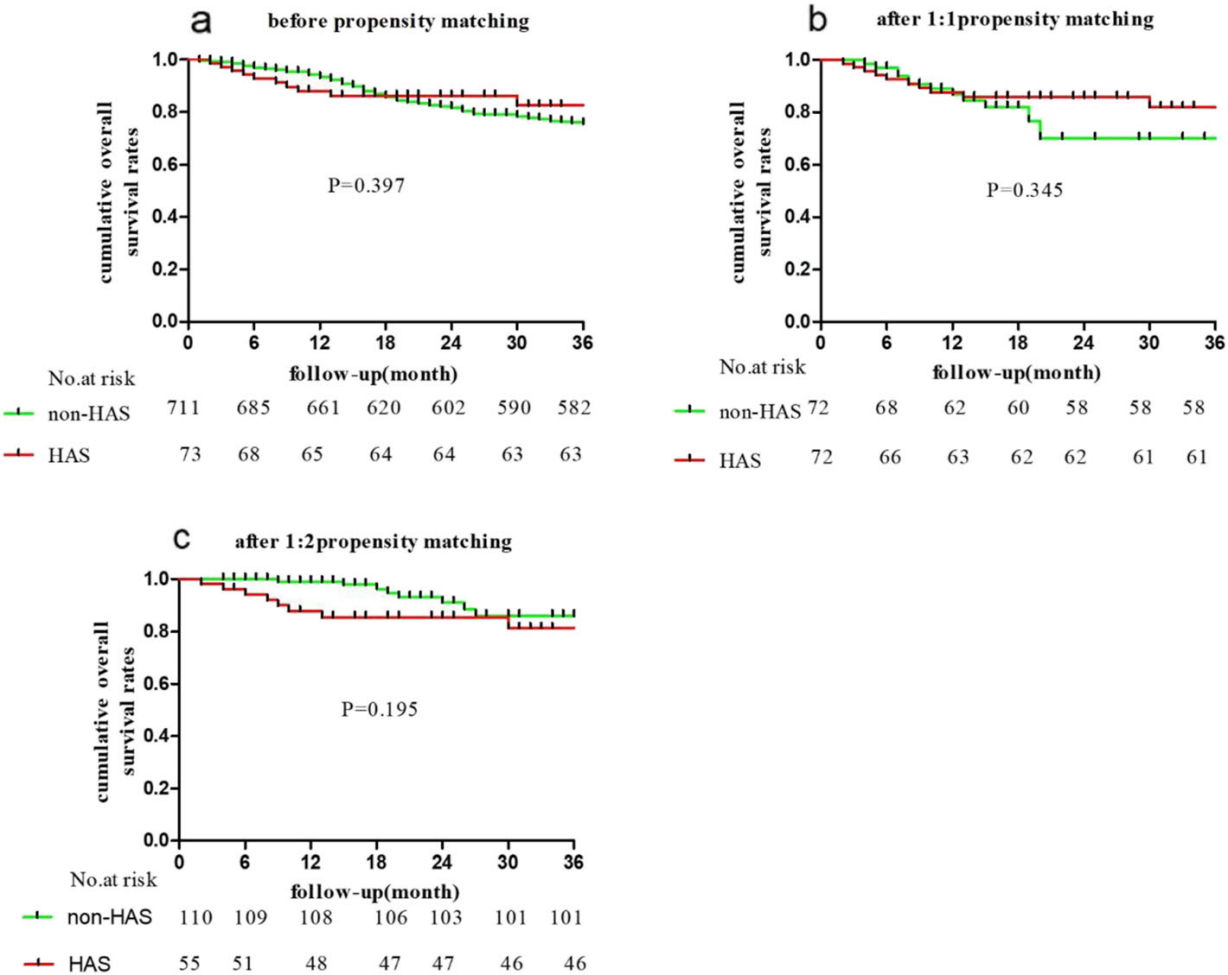
Kaplan-Meier survival plots were made by using GraphPad Prism 5

In our analysis, we found that OS was not significantly different between the HAS group and the non-HAS group (Fig. 2). The median follow-up time of the pre-PSM cohort was 22.0 months (rang = 0 to 97 months). The median follow-up time was 15.0 months (rang = 0 to 97 months) after 1:1 PSM. The median follow-up time was 21.0 months (range = 0 to 97 months) after 1:2 PSM. Among the 784 patients (13 patients with distant metastasis were excluded from 797 patients) in our study, the 1-, 2-, and 3-year survival rates of non-HAS patients were 92.6, 81.1, and 75.0%, and those of HAS patients were 87.9, 86.2, and 82.6%, respectively. Among the one-to-one nearest-neighbour matched pairs of patients, the 1-, 2-, and 3-year survival rates of non-HAS patients were 94.4, 86.5, and 82.3%, and those of HAS patients were 92.9, 86.3 and 84.5%, respectively. Among one-to-two nearest-neighbour matched pairs of patients, the 1-, 2-, and 3-year survival rates of non-HAS patients were 98.1, 98.1, and 96.9%, and those of HAS patients were 90.3, 83.9 and 79.9%, respectively.

### Risk factors for prognosis

Among the 784 patients, univariate analysis showed that the tumour location, surgery type, vascular invasion, T and N stage, the levels of CEA and CA19–9, EGFR expression and neoadjuvant chemotherapy were significantly associated with OS. Among the one-to-one nearest-neighbour matched pairs of patients, T and M stage, EGFR expression and neoadjuvant chemotherapy were found to be significantly related to OS. Among the one-to-two propensity-matched pairs of patients, T and M stage, level of CEA and EGFR expression were significantly associated with OS (Table 2).

**Table 2:** Univariate analyses of OS used the Kaplan-Meier approach

Factors (k-m)	Before propensity matching p value*	After 1:1 propensity matching p value	After 1:2 propensity matching p value
	OS	OS	OS
GC types^a^	0.410	0.345	0.19
Age	0.368	0.277	0.446
Sex	0.982	0.584	0.322
Location	0.001	0.903	0.555
Surgery type	<0.001	0.530	0.471
Vascular invasion	<0.001	0.120	0.101
T	<0.001	0.001	0.013
N	<0.001	0.201	0.431
M	-	<0.001	<0.001
CEA	0.006	0.066	<0.002
CA199	<0.001	0.552	0.312
EGFR	<0.001	<0.001	0.007
HER2	0.380	0.397	0.644
KI-67	0.187	0.067	0.258
Neoadjuvant chemotherapy	0.002	0.043	0.080

^a^ GC types: hepatoid adenocarcinoma of the stotmach and non-hepatoid adenocarcinoma of the stomach

*Log Rank (Mantel-Cox)

Among the 784 patients, multivariate analysis identified prognostic factors including T and N stage, EGFR and neoadjuvant chemotherapy. There was no statistical difference of OS between HAS and non-HAS by using multivariable Cox regression models given the following covariance: age, tumor location, surgery type, vascular invasion, T and N stage, the level of CEA and CA199, EGFR and neoadjuvant chemotherapy. (P = 0.619) (Table 3a). Among the one-to-one nearest-neighbour matched pairs of patients, the univariate analysis identified some factors significantly related to OS, including the T stage, M stage, the level of CEA, EGFR and neoadjuvant chemotherapy. There was no statistical difference of OS between HAS and non-HAS by using multivariable Cox regression models given the following covariance: age, vascular invasion, TNM stage, the level of CEA and CA199, EGFR and neoadjuvant chemotherapy. (*p* = 0.841) (Table 3b). Among the one-to-two nearest-neighbour matched pairs of patients, M stage and the level of CEA associated with OS. There was no statistical difference of OS between HAS and non-HAS by using multivariable Cox regression models given the following covariates: age, vascular invasion, TNM stage, the level of CEA and CA199, EGFR and neoadjuvant chemotherapy. (p = 0.098) (Table 3c).

**Table 3:** Multivariable survival analysis to identify factors predicting OS by using multivariable Cox regression models a Before propensity matching

a Before propensity matching			
Factor	HR	95%CI	P value
GC type			
non-HAS	-	-	-
HAS	1.193	0.596 -2.386	0.619
Age (yr)			0.463
<45	-	-	-
60>age≥45	1.113	0.621-1.993	0.720
≥60	1.335	0.752-2.372	0.324
Location			0.978
U	-	-	-
M	1.000	0.628-1.594	0.999
L	1.031	0.573-1.857	0.918
T	1.304	0.392-4.334	0.665
Surgery type			0.152
PG	-	-	-
DG	1.096	0.137-8.782	0.931
TG	1.805	0.242-13.456	0.564
TGC	4.924	0.407-59.619	0.210
Vascular invasion			
no	-	-	
yes	1.145	0.771-1.701	0.503
T			<0.001
Tis,T0,T1,T2	-	-	-
T3	1.930	0.939-3.967	0.074
T4	3.518	1.756-7.050	<0.001
N			0.001
N0	-	-	-
N1	1.945	0.983-3.847	0.056
N2	2.133	1.070-4.252	0.031
N3	3.579	1.839-6.966	<0.001
CEA (ng/ml)			
≤5	-	-	-
>5	1.460	0.992-2.151	0.055
CA199 (u/ml)			
≤37	-	-	-
>37	1.514	0.998-2.297	0.051
EGFR			0.046
-	-	-	-
+	1.342	0.788-2.286	0.279
++	0.766	0.399-1.468	0.422
+++	0.786	0.411-1.503	0.467
Neoadjuvant chemotherapy			
no	-	-	-
yes	1.724	1.123-2.646	0.013
b After 1:1 propensity matching			
Factor	HR	95%CI	P
GC type			
non-HAS	-	-	-
HAS	1.114	0.390-3.183	0.841
Age (yr)			0.158
<45	-	-	-
60>age≥45	1.103	0.120-10.169	0.931
≥60	2.716	0.322-22.934	0.359
Vascular invasion			
no	-	-	-
yes	4.633	0.936-22.942	0.060
T			0.049
Tis,T0,T1,T2	-	-	-
T3	3.779	0.674-21.186	0.131
T4	8.435	1.408-50.544	0.020
N			0.241
N0	-	-	-
N1	0.113	0.014-0.921	0.042
N2	0.233	0.033-1.668	0.147
N3	0.198	0.024-1.659	0.136
M			
M0	-	-	-
M1	7.354	1.760-30.737	0.006
CEA (ng/ml)			
≤5			
>5	3.123	1.276-7.643	0.013
CA199 (u/ml)			
≤37			
>37	4.275	0.759-24.071	0.099
EGFR			0.030
-	-	-	-
+	0.115	0.010-1.347	0.085
++	0.028	0.002-0.375	0.007
+++	0.101	0.009-1.159	0.066
Neoadjuvant chemotherapy			
no	-	-	-
yes	3.816	1.339-10.878	0.012
c After 1:2 propensity matching			
Factor	HR	95%CI	P
GC type			
non-HAS	-	-	-
HAS	2.579	0.839-7.925	0.098
Age (yr)			0.306
<45	-	-	-
60>age≥45	5.503	0.159-190.915	0.346
≥60	9.580	0.304-301.867	0.199
Vascular invasion			
no	-	-	-
yes	2.597	0.472-14.281	0.273
T			0.207
Tis,T0,T1,T2	-	-	-
T3	5.591	0.775-40.367	0.088
T4	6.032	0.732-49.721	0.095
N			0.640
N0	-	-	-
N1	0.637	0.072-5.636	0.685
N2	0.355	0.036-3.515	0.376
N3	0.912	0.111-7.474	0.932
M			
M0	-	-	-
M1	51.522	2.359-1125.341	0.012
CEA (ng/ml)			-
≤5	-	-	
>5	8.116	2.294-28.719	0.001
CA199 (u/ml)			
≤37			
>37	0.000	0.000-	0.983
EGFR			0.214
-	-	-	-
+	0.150	0.014-1.587	0.115
++	0.094	0.009-0.929	0.043
+++	0.136	0.019-0.996	0.050
Neoadjuvant chemotherapy			
no	-	-	-
yes	2.717	0.729-10.131	0.137

## Discussion

HAS comprises polygonal cells arranged in solid or trabecular form, similar to that in hepatocellular carcinoma [12, 22]. Many researchers supported that the common embryos of the stomach and liver originated from the foregut and may evolve through genetic progression and/or genetic differences [23, 24]. At present, there were two views on the prognosis of HAS and non-HAS. The majority of studies showed that HAS had a distinctly poorer prognosis than non-HAS [9, 10, 25]. However, a few reports suggested that HAS did not have a poorer prognosis. Although many researchers explored the clinical characteristics of HAS, there was still no unified standard for its diagnosis and treatment, and most of them were case reports [26]. In addition, owing to inadequate understanding of HAS and clinicians and pathologists did not pay much attention to it [21]. Therefore, it may be clinically difficult to draw a consistent conclusion of the prognostic impact of HAS. Certainly because of this, our study aimed to further elucidate whether HAS had a worse prognosis than non-HAS using a larger number of patients and whether HAS can benefit from radical surgery.

We used the propensity score matching method to eliminate the bias between HAS and non-HAS patients and then compared their prognoses. Our study showed that there was no significant difference in postoperative OS between HAS and non-HAS patients within 3 years after radical surgical resection, which is contrary to the majority of findings. The research from Liu et al. showed a significantly different prognosis between HAS and non-HAS [27]. The 1-, 3-, and 5-year survival rates of HAS and non-HAS (without AFP production) were 30, 13, and 9% and 95, 57, and 38%, respectively [27]. In their research, the incidence of liver metastasis was 75.6% (34/45), including 8.9% synchronous and 73.2% (30/41) metachronous liver metastasis [27]. However, in the study by Cheon SH et al., among 10,259 patients diagnosed with gastric adenocarcinoma and 58 patients had live-only metastases after gastric resection [28]. Our research had seven HAS patients with postoperative liver metastasis (7/73). Therefore, we boldly speculated that the patients of HAS may had a higher risk of liver metastases [7, 29] than non-HAS, and the occurrence of liver metastasis contributed to the poorer prognosis of HAS, which was consistent with the findings of some reports [12, 30, 31]. It was worth mentioning that the prognosis of patients with higher serum levels of AFP was poorer than that of patients with lower serum levels of AFP (< 500 ng/ml) [7]. Essentially, there remained no clear reasons for the poorer OS of HAS. Some researchers believe that HAS produced alpha-1 antitrypsin (AAT) and/or alpha-1 antichymotrypsin (ACT) and AFP, which enhanced invasiveness and affect immunosuppressive properties [12, 32, 33]. However, a few studies were consistent with ours. Wang et al., demonstrated that patients with HAS who underwent radical surgery had a 5-year survival rate of 41.1% [7]. Augustin G reported that a 72-year-old man was diagnosed with HAS underwent gastrectomy and splenectomy. He was still alive 24 months after surgery without distant metastasis [34]. Giustozzi G et al. reported that a HAS patient with radical surgery who underwent chemotherapy was still alive and disease-free (with a 52-month follow-up) [35]. Therefore, radical surgery and adjuvant chemotherapy may have a positive impact on the therapeutic effect [36, 37].

In our research, the univariate analysis suggested that both T and M stage were related to the OS of gastric adenocarcinoma in the groups of data before and after PSM, but the N stage had no significant relationship with prognosis in the data after propensity score matching. This finding may be attributed to the limited number of samples after PSM. In the multivariate analysis, before PSM, T and N stage, the level of EGFR, and neoadjuvant chemotherapy were independent risk factors affecting the OS of HAS and non-HAS patients. T stage, the level of CEA and EGFR and neoadjuvant chemotherapy were independent risk factors affecting the OS of HAS and non-HAS patients in the 1:1 propensity matching. The level of CEA was an independent risk factor that affected the OS of patients after radical surgery in 1:2 propensity matching. Although distant metastasis (M1) was a significant predictor of OS after PSM, we have less data on distant metastasis, it was not convincing.

Our study had several limitations. First, it was retrospective and enrolled patients in a single institutional cohort. Second, we did not include information about the patient’s postoperative chemotherapy in our study. However, we usually decided whether to give chemotherapy to patients according to their postoperative pathological results. Lastly, the follow-up time was not long enough to assess long-term prognosis. Despite these limitations, a relatively large number of patients and rigorous statistical methods made our results convincing.

## Conclusion

There was no statistically significant difference in the overall survival time between patients with HAS and non-HAS after radical surgery and adjuvant chemotherapy. Under the condition that patients with HAS could tolerate the surgery, the choice of surgery indications and methods were the same as that of non-HAS, radical surgery was the best choice for HAS patients.

## Supplementary information

**Additional file 1.**

**Additional file 2.**

## Data Availability

The datasets used and/or analyzed during the current study are available by contacting kai zhou by email (zhoukai0615@pku.edu.cn) on a reasonable request.

## Abbreviations

HAS: Hepatoid adenocarcinoma of the stotmach
non-HAS: Non-hepatoid adenocarcinoma of the stomach
OS: The overall survival time
PSM: Propensity score matching
GC: Gastric carcinoma
CEA: Carcinoembryonic antigen;
CA199: Carbohydrate antigen 19–9
MRI: Magnetic resonance imaging
CT: Computed tomography
AAT: Alpha-1 antitrypsin
ACT: Alpha-1 antichymotrypsin
IHC: Immunohistochemistry
