# Supplementary material for: The prognosis of hepatoid adenocarcinoma of the stomach: a propensity score-based analysis
Source: BMC Cancer. 2020 Jul 17;20:671. doi: 10.1186/s12885-020-07031-9 (PMC7368673; doi:10.1186/s12885-020-07031-9)
Supplement: Supplementary file 1 — Additional file 1. [file 12885_2020_7031_MOESM1_ESM.pdf]

Additionalfile1:Table1 Before excluding the patients with M1, clinicopatholodical characteristics of patients with HAS and Non-HAS treated with radical gastrectomy.

Factors	Before propensity matching		
	Non-HAS<br>n=722<br>No.(%)	HAS<br>N=75<br>No.(%)	P value
Sex(M/F)	517/205	61/14	0.072
Age (yr)			0.785
＜45	81(11.2%)	7(9.3%)	
60＞age≥45	282(39.1%)	32(42.7%)	
≥60	359(49.7%)	36(48.0%)	
Location			0.762
U	217(30.1%)	25(33.3%)	
M	130(18.0%)	9(12%)	
L	365(50.6%)	41(54.7%)	
T	10(1.4%)	0(0.0%)	
Surgery type			0.170
PG	9(1.2%)	2(2.7%)	
DG	354(49.0%)	41（54.7%）	
TG	356(49.3%)	32（42.7%）	
TGC	3(0.4%)	0（0.0%）	

Vascular invasion			0.272
no	327（45.3%）	292（38.7%）	
yes	395（54.7%）	46(61.3%)	
T			0.001
Tis,T0，T1,T2	171(23.7%)	21（28.0%）	
T3	263（36.4%）	40（53.3%）	
T4	288（39.9%）	14（18.7%）	
N			0.229
N0	221（30.6%）	11(14.7%)	
N1	155（21.5%）	24(32.0%)	
N2	146（20.2%）	23（30.7%）	
N3	200（27.7%）	17（22.7%）	
M			0.791
M0	711（98.5%）	73（97.3%）	
M1	11（1.5%）	2（2.7%）	
EGFR			<0.001
-	64（8.9%）	2（2.7%）	
+	269（37.3%）	7（9.3%）	
++	196（27.1%）	37（49.3%）	
+++	193（26.7%）	29（38.7%）	

Ki-67			0.003
0-25%	67（9.3%）	5（6.7%）	
26-50%	169（23.4%）	6（8.0%）	
51-75%	212（29.4%）	25（33.3%）	
76-100%	274（38.0%）	39（52.05）	
CEA（ng/ml）			<0.001
≤5	582（80.6%））	45（60.0%）	
>5	140(19.4%)	30（40.0%）	
CA199（u/ml）			0.022
≤37	614（85.0%）	71（94.7%）	
>37	108（15%）	4（5.3%）	
Her-2			0.010
-/+	541（74.9%）	44（58.7%）	
+++	55（7.6%）	10（13.3%）	
++	126（17.5%）	21（28.0%）	
neoadjuvant chemotherapy			0.003
no			
yes	637（88.2%）	57（76.0%）	
	85（11.8%）	18（24.0%）	
